# Function of Drosophila Synaptotagmins in membrane trafficking at synapses

**DOI:** 10.1007/s00018-021-03788-9

**Published:** 2021-02-22

**Authors:** Mónica C. Quiñones-Frías, J. Troy Littleton

**Affiliations:** 1 Department of Biology, The Picower Institute for Learning and Memory, Massachusetts Institute of Technology (MIT), Bldg. 46-3243, 43 Vassar St., Cambridge, MA 02139 USA; 2 Department of Brain and Cognitive Sciences, The Picower Institute for Learning and Memory, Massachusetts Institute of Technology (MIT), Bldg. 46-3243, 43 Vassar St., Cambridge, MA 02139 USA; 3 Department of Biology, Brandeis University, Waltham, MA USA

**Keywords:** Drosophila, Synapse, Neurotransmitter release, Synaptic vesicle, Synaptotagmin, Exocytosis

## Abstract

The Synaptotagmin (SYT) family of proteins play key roles in regulating membrane trafficking at neuronal synapses. Using both Ca^2+^-dependent and Ca^2+^-independent interactions, several SYT isoforms participate in synchronous and asynchronous fusion of synaptic vesicles (SVs) while preventing spontaneous release that occurs in the absence of stimulation. Changes in the function or abundance of the SYT1 and SYT7 isoforms alter the number and route by which SVs fuse at nerve terminals. Several SYT family members also regulate trafficking of other subcellular organelles at synapses, including dense core vesicles (DCV), exosomes, and postsynaptic vesicles. Although SYTs are linked to trafficking of multiple classes of synaptic membrane compartments, how and when they interact with lipids, the SNARE machinery and other release effectors are still being elucidated. Given mutations in the SYT family cause disorders in both the central and peripheral nervous system in humans, ongoing efforts are defining how these proteins regulate vesicle trafficking within distinct neuronal compartments. Here, we review the Drosophila SYT family and examine their role in synaptic communication. Studies in this invertebrate model have revealed key similarities and several differences with the predicted activity of their mammalian counterparts. In addition, we highlight the remaining areas of uncertainty in the field and describe outstanding questions on how the SYT family regulates membrane trafficking at nerve terminals.

## Synaptic communication and the synaptic vesicle release machinery

The nervous system relies on regulated secretion of neurotransmitters to meditate synaptic communication between neurons [1, 2]. Synaptic transmission typically occurs at specialized release sites in presynaptic terminals known as active zones (AZs). Highly conserved AZ scaffold proteins concentrate voltage-gated Ca^2+^ channels and synaptic vesicles (SVs) to position the release machinery near clustered neurotransmitter receptors in the postsynaptic membrane [3–14]. During action potential propagation along the axon, membrane depolarization triggers the opening of voltage-gated Ca^2+^ channels and influx of extracellular Ca^2+^ to trigger SV fusion at release sites [2, 15–20]. SV exocytosis is a highly stochastic process at individual AZs, with the probability of an individual fusion event varying over a wide range depending on the neuronal population [21–23]. The evoked response recorded postsynaptically represents the probability of SV fusion events that occur over a population of individual release sites.

**Fig. 1:**
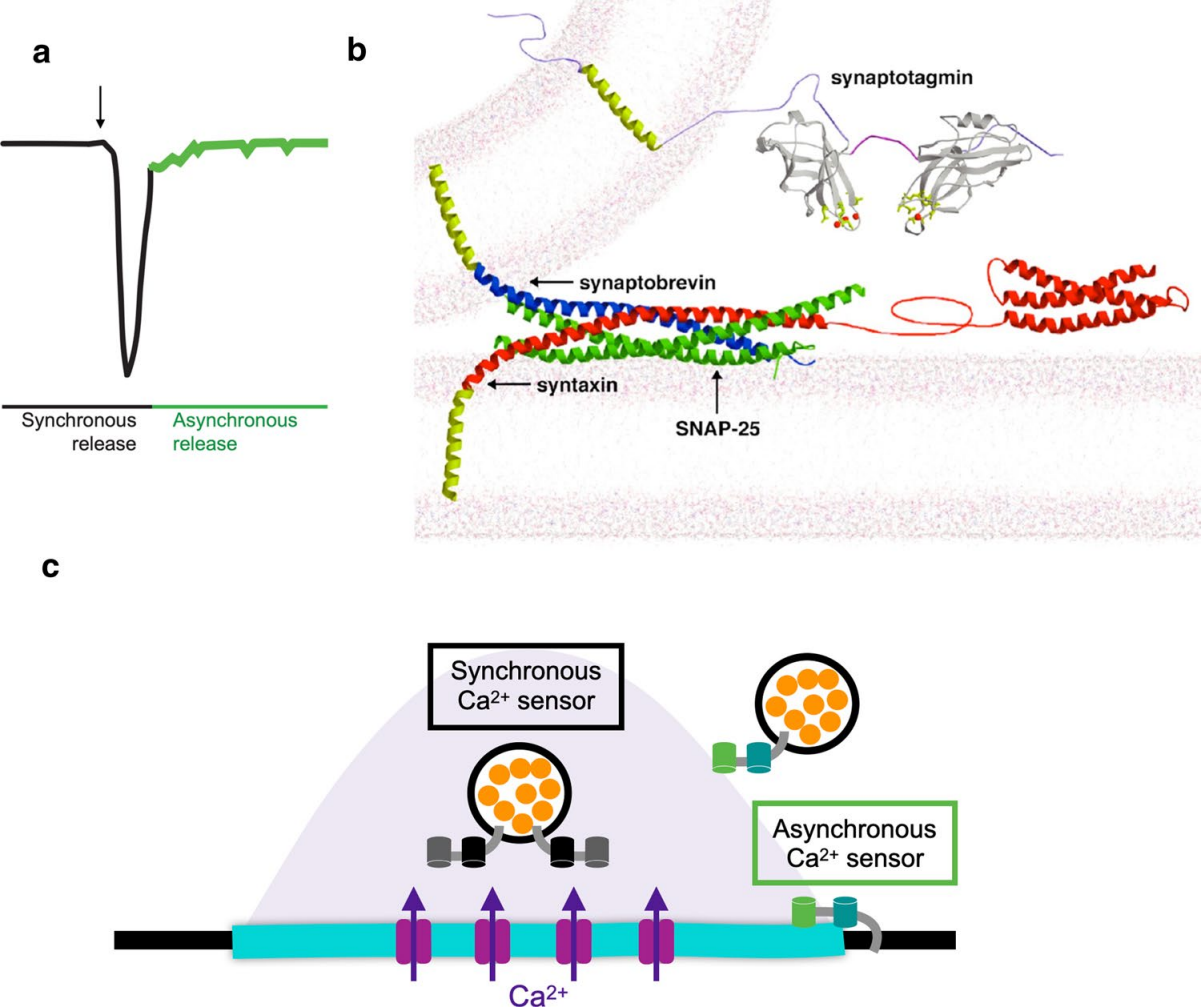
Regulation of synchronous and asynchronous SV fusion. **a** Model depicting phases of synchronous and asynchronous release after nerve stimulation (arrow). **b** Structure of the fusion machinery that bridges the SV and plasma membrane, with SYT1 (grey) and the SNARE complex (Syntaxin—red; SNAP25—green; Synaptobrevin—blue). **c** Model of synchronous and asynchronous release in relation to presynaptic Ca^2+^ entry (shaded). Following Ca^2+^ influx, SVs fuse during a synchronous phase at AZs that occurs within milliseconds. SYT1 acts as the Ca^2+^ sensor for synchronous release and resides on SVs. A slower asynchronous component can last for hundreds of milliseconds and include fusion of SVs farther from release sites. SYT7 has emerged as a candidate for the asynchronous Ca^2+^ sensor with higher affinity than SYT1 for Ca^2+^ binding. The role of SYT7 is still controversial, with studies in Drosophila suggesting it controls SV availability and fusogenicity

The timing for single SV fusion events can occur over a relatively broad temporal window of ~ 1–200 ms at individual AZs following an action potential (Fig. 1a). Release kinetics have been loosely classified into two distinct phases termed synchronous and asynchronous release [24]. The synchronous phase accounts for the majority of neurotransmitter release at most synapses, with SV fusion events decaying within several milliseconds after presynaptic Ca^2+^ influx. The asynchronous phase (also known as the delayed response) is less pronounced at most synapses but becomes more robust during high frequency nerve stimulation. In this slower component of release, individual SV fusion events occur within several hundred milliseconds across the AZ population [19, 25, 26]. In addition to the two phases of evoked release, single SVs can fuse spontaneously (termed minis) in the absence of nerve stimulation [23, 26–28].

All three pathways for SV release (synchronous, asynchronous, spontaneous) require a highly conserved fusion machinery that controls the regulated assembly of a four stranded coiled-coil SNARE complex that bridges the SV and presynaptic membranes [29–31]. This complex includes the v-SNARE Synaptobrevin on the SV, and the t-SNAREs Syntaxin and SNAP-25 on the presynaptic plasma membrane (Fig. 1b). Multiple SNARE chaperones, including the UNC13 and UNC18 families, position and regulate the timing of the highly energetic assembly of the SNARE alpha-helices during the SV cycle [2, 32–35]. Current models suggest the coiled-coil SNARE bundle partially assembles at the SV-plasma membrane interface prior to fusion in a *cis*-conformation where the transmembrane domains of Synaptobrevin and Syntaxin remain separated in the SV and plasma membrane, respectively. The assembly and arrest of SNARE zippering at this intermediate state is regulated by the cytosolic SNARE-binding protein Complexin (CPX), which is hypothesized to provide a clamping brake on fusion until Ca^2+^ entry occurs [36–43]. The SYT1 family of SV Ca^2+^ sensors binds Ca^2+^ ions through the action of a cluster of negatively charged aspartate residues present in loop structures that emerge from its two C2 domains (Fig. 1b). The Ca^2+^-bound loops of SYT1 interact with negatively charged lipid headgroups in the presynaptic membrane. This interaction alters local lipid structure and helps trigger full zippering of the SNARE complex into a trans-SNARE state where the transmembrane domains of the SNARE proteins reside together in the fused membrane [2, 40, 44–46]. In addition to activating release, SYT1 also inhibits spontaneous fusion through a clamping mechanism similar to the role of CPX [47–55]. Together, these proteins act to drive full collapse of the SV into the plasma membrane during presynaptic Ca^2+^ influx to exocytose neurotransmitters through the fast synchronous pathway [24, 51, 56–64].

The asynchronous release pathway uses a similar fusion machinery with the major exception of not requiring SYT1 (Fig. 1c). Several mechanisms have been proposed that differentiate asynchronous and synchronous release [16, 24, 65–74], including distinct Ca^2+^ sensors, heterogeneity in SV protein content, SV distance from Ca^2+^ channels, distinct Ca^2+^ entry pathways, and regulation of Ca^2+^ extrusion and buffering. Asynchronous release is enhanced in *Syt1* mutants [51, 57, 75], suggesting Ca^2+^ can activate this slower pathway through a distinct Ca^2+^ sensor(s). Manipulations of SYT7 change the amount of release occurring through the slower asynchronous SV fusion pathway, but whether the protein functions as a Ca^2+^ sensor for the fusion process itself or modifies Ca^2+^-dependent SV availability is unclear [58, 76–84]. Following fusion of SVs through either pathway, α-SNAP binding to *trans*-SNARE complexes in the plasma membrane recruits the AAA ATPase NSF to disassemble the complex and recharge individual SNAREs for additional rounds of release [29, 85–90]. NSF can also disassemble trans-SNARE complexes present on the SV that escape endocytosis control, releasing free v-SNAREs to form productive *cis*-SNARE complexes required for fusion [91]. Following endocytosis, SNARE chaperones and the priming machinery reposition fusogenic SVs at AZs for additional cycles of release.

In the current review, we discuss models for how the SYT family regulates membrane trafficking at synapses. In particular, we focus on studies performed in Drosophila that examine the function of the three most abundant SYT isoforms (SYT1, SYT4, and SYT7) that are found at most synapses. Similar to Drosophila, the homologs of these three SYT isoforms are among the most abundant members in mammals [84]. SYT1 and SYT7 have been shown to regulate SV trafficking in both systems, while SYT4 has been linked to presynaptic exosomes and postsynaptic retrograde signaling in Drosophila. Electrophysiology, imaging and structure–function studies have provided insights into how SYT1, SYT4, and SYT7 regulate synaptic communication in vivo. We review this data, as well as examine what is known about the remaining family members. Finally, we highlight recent studies identifying mutations in SYT family proteins in human neurological disorders and describe work on potential pathological mechanisms using Drosophila models.

## Analyzing synaptic transmission at Drosophila neuromuscular junctions

**Fig. 2:**
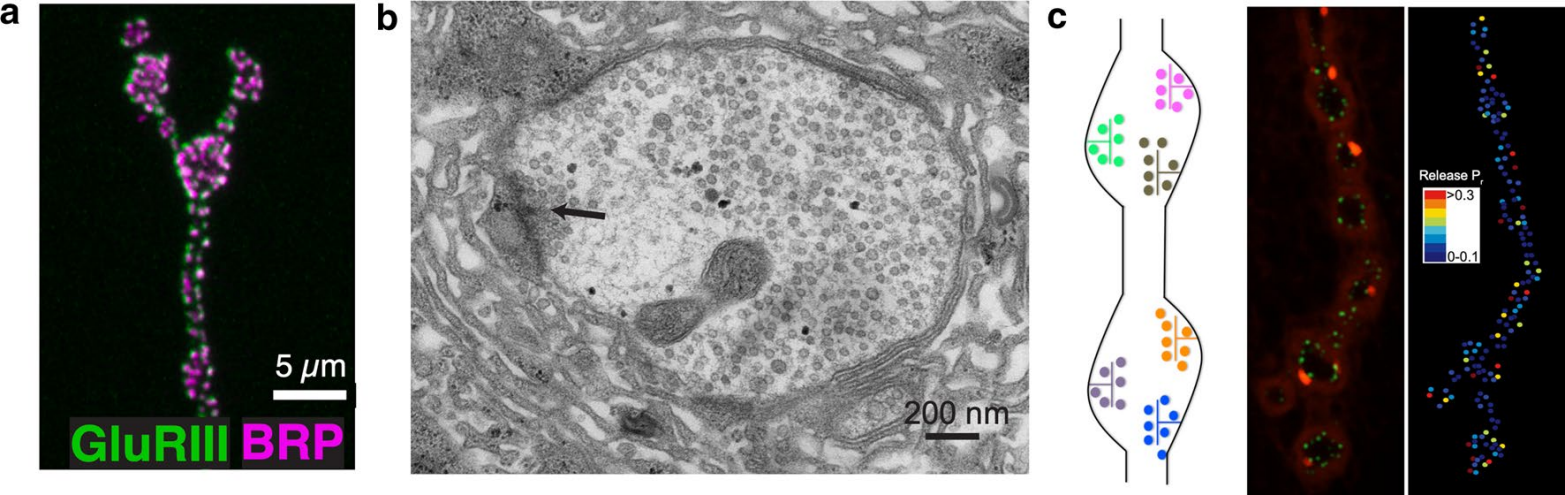
The Drosophila larval NMJ as a model for synaptic function. **a** Immunolabeling of the nerve terminal showing AZs (anti-BRP, magenta) and post-synaptic densities (anti-Glutamate Receptor III staining, green) at a larval NMJ. **b** EM of a single synaptic bouton with an AZ T-bar denoted (arrow). **c** The left panel shows a model of synaptic boutons with multiple individual AZs (left). The middle panel shows an NMJ expressing the presynaptic Ca^2+^ channel (Cac-GFP, green) and evoked SV release events (red) visualized with a modified jRGECO Ca^2+^ indicator expressed postsynaptically. The right panel shows quantal imaging of evoked release probability at the same NMJ, revealing heterogeneity in AZ strength as noted on the color-coded heat map. **a** Modified from [105], **b** modified from [82], and **c** modified from [22]

Electrophysiological and imaging analysis at Drosophila 3rd instar larval neuromuscular junction (NMJ) synapses has proven highly effective for dissecting the roles of SYTs in synapse biology. At this connection, motoneurons form glutamatergic synapses onto muscles, with motor axons containing tens of en passant presynaptic boutons with hundreds of individual AZs highlighted by a T-bar filamentous structure at the center (Fig. 2a, b) [14]. SV release from motoneurons can be measured using electrophysiological recordings of synaptic currents from the muscle or by optical imaging of postsynaptic fusion events. Individual AZs at the NMJ are aligned to discrete postsynaptic glutamate receptor (GluR) fields. This arrangement facilitates use of modified GCaMPs expressed postsynaptically to image SV fusion events by visualizing spatially localized Ca^2+^ influx following GluR opening (Fig. 2c) [22, 23, 92–94]. This toolkit makes the Drosophila NMJ one of the only models where every SV fusion event can be imaged and assigned to single AZs, greatly increasing the spatial resolution of SV release compared to electrophysiology alone. These imaging approaches demonstrate single quanta are released at AZs following an action potential, as multi-vesicular release at individual sites is rare [22, 23, 92–95]. Similar to other systems, AZs formed by a single Drosophila motoneuron display heterogeneity in SV release probability (*P*_r_), with a small population of strong AZs present amongst many weaker ones (Fig. 2c). Highlighting the stochastic nature of the release process, the average AZ *P*_r_ is ~ 0.07 at the NMJ, indicating most AZs release a SV less than 10% of the time in response to single action potentials [22]. This quantal imaging approach has been applied to *Syt1* and *Syt7* mutants to examine SV release at individual AZs.

## The Synaptotagmin superfamily

**Fig. 3:**
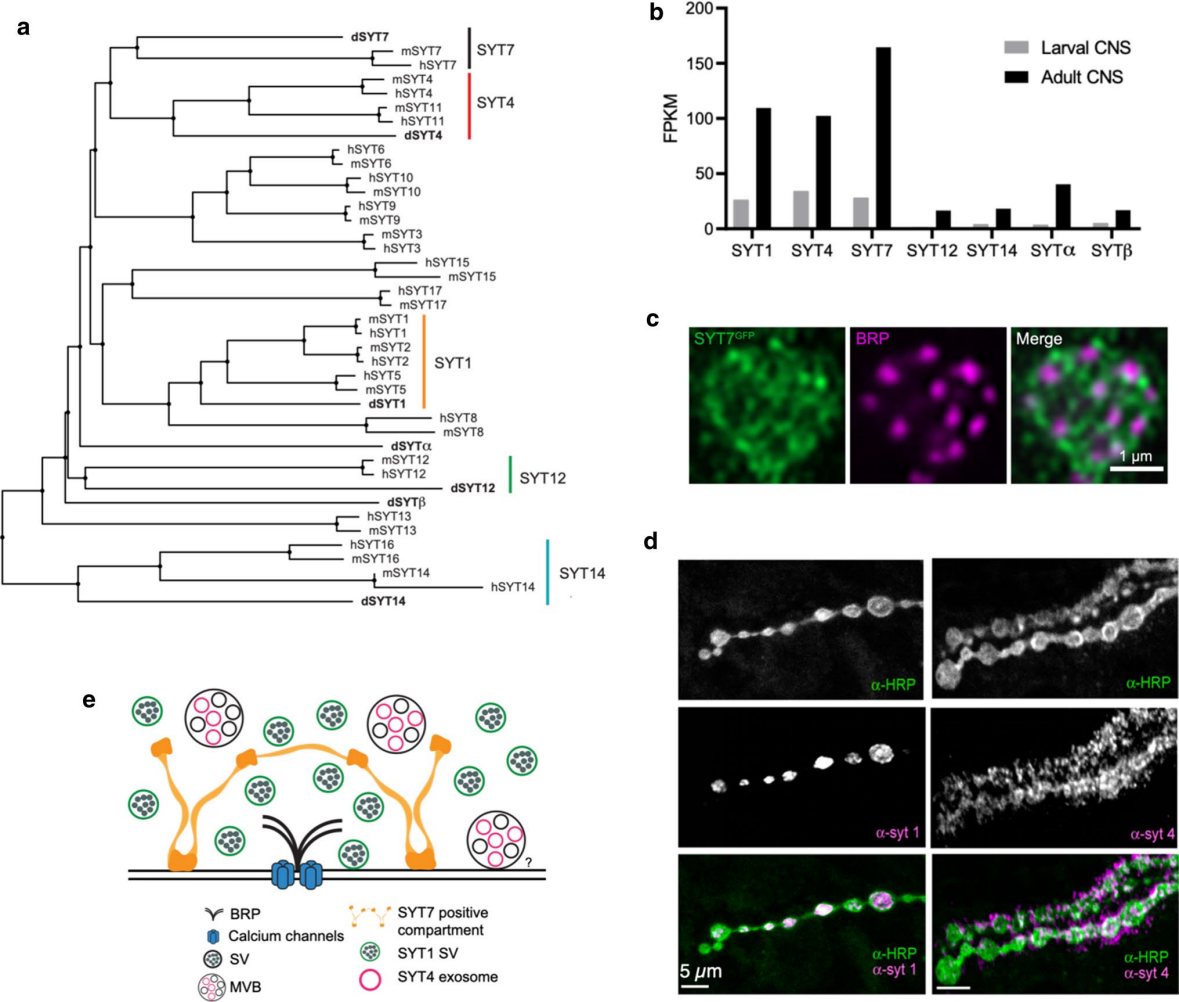
Conservation, abundance and localization of Drosophila SYTs. **a** Phylogenetic tree of SYT homologs in *Drosophila melanogaster* (d), *Mus musculus* (m), and *Homo sapiens* (h). The SYT1, SYT4, SYT7, SYT12, and SYT14 subfamilies are highlighted. The sequences of each SYT were extracted from NCBI and the tree was generated using neighbor clustering algorithm. **b** Expression level of Drosophila *Syt* genes in larval and adult brain using RNAseq. **c** Expression of endogenously CRISPR-tagged SYT7-GFP compared to the AZ protein BRP in a single larval NMJ bouton. SYT7 surrounds AZs and localizes to an interconnected tubular membrane compartment within the peri-AZ. **d** Localization of SYT1 to SVs and SYT4 to postsynaptic puncta at the larval NMJ using immunocytochemistry. The motor axon is stained with anti-HRP (green). **e** Model of the subcellular localization of Drosophila SYTs. SYT1 is attached to SVs and triggers synchronous SV fusion. SYT7 localizes to an internal peri-AZ compartment and negatively regulates SV re-entry into the readily releasable pool and SV fusogenicity. SYT4 localizes to presynaptic exosomes that are released from multi-vesicular bodies (MVBs) to transfer the protein to the postsynaptic compartment where it mediates retrograde signaling. The graph in panel b was generated by plotting gene expression levels in the CNS of 7-day-old males reported in [368], panel c was modified from [100], and panel d was modified from [82]

SYTs are conserved family of membrane-trafficking proteins containing a single transmembrane domain and two paired cytosolic C2 domains (Figs. 1b, 3). Their expression is largely restricted to the nervous system, though a few isoforms participate in a smaller subset of trafficking pathways in non-neuronal cells. Genes encoding SYT proteins are not found in bacterial or yeast genomes, indicating intracellular membrane fusion events like ER to Golgi trafficking do not require the function of this protein family. The first members of the SYT family emerged during evolution in the placozoans, a primitive branch of multi-cellular metazoans that lack neurons. Homologs of SYT1 and SYT7 are encoded in the *Trichoplax adhaerens* genome, suggesting a non-neuronal origin of SYTs during a period when multicellular communication was emerging [96, 97]. The SYT family expanded during invertebrate evolution, and the Drosophila genome encodes seven distinct SYT proteins. With genome duplications in vertebrates, greater diversification occurred and 17 SYT family members are encoded in the human genome [96]. Many SYT proteins can be grouped into functional orthologs based on sequence similarity across evolution, while other isoforms are more divergent without clear orthologs between invertebrate and vertebrate proteins (Fig. 3a).

SYTs can be subdivided into whether or not they are likely to bind Ca^2+^ based on conservation of negatively charged aspartate residues within their C2 domains that mediate this interaction [98]. For the seven Drosophila SYT homologs, five are predicted to bind Ca^2+^ (SYT1, SYT4, SYT7, SYTα, SYTβ), while two lack conserved Ca^2+^ binding aspartate residues (SYT12, SYT14) [99, 100]. Three Drosophila SYT isoforms are highly expressed in neurons (SYT1, SYT4, SYT7) based on in situ mRNA expression and RNAseq analysis (Fig. 3b) [100]. SYT1 is homologous to mammalian SYT1, SYT2, and SYT9 proteins, all of which reside on SVs (Fig. 3c) and function as Ca^2+^ sensors driving fast synchronous SV fusion in specific neuronal populations [57, 60, 61, 63, 101]. Drosophila *Syt1* null mutants generally die as embryos or during early larval development [63, 102], though some can survive to adulthood at low frequency when cultured directly on food where minimal movement is required [103]. These surviving adults are completely ataxic and die within several days. SYT7 is encoded by single gene in both Drosophila and mammals and has been linked to asynchronous SV release [80, 82, 84]. The Drosophila homolog resides on a tubular membrane compartment present within the peri-AZ, a synaptic domain implicated in endocytosis and protein sorting (Fig. 3d). Drosophila SYT4 is homologous to mammalian SYT4 and SYT11 and is transferred to the postsynaptic compartment from exosomes (Fig. 3b), where it functions in retrograde signaling [97, 100, 104–107]. Mutations in Drosophila *Syt4* and *Syt7* are viable and fertile as adults [82, 97, 104, 105], though no studies to date have examined potential roles in synaptic plasticity and learning in adult animals. SYT12 and SYT14 are more divergent in sequence, but appear orthologous to mammalian SYT12 and SYT14 [99]. These isoforms are expressed at low levels in Drosophila and little is known about their function. SYTα and SYTβ are present on dense core vesicles (DCVs) in neuroendocrine neurons and are presumed to function as DCV Ca^2+^ sensors in Drosophila [100, 108–110]. Their precise relationship to specific mammalian SYT isoforms is unclear. No mutants in these final four SYT isoforms have been described. Beyond these seven SYT family members in Drosophila, there are multiple genes encoding proteins that contain C2 domains that are orthologs of other synaptic and non-synaptic proteins that will not be discussed. These include the ER residents extended SYT (ESYT) and multiple C2 domain protein (MCTP) family members [111–114], homologs of the synaptic proteins Rabphilin, Rim, and Munc13 [99, 115, 116], as well as homologs of mammalian Otoferlin (Misfire) [117] and Granulophilin (Bitesize) [118]. In contrast to mammals, the Drosophila genome does not encode a homolog of the DOC2 family, a group of SYT-like proteins with two cytosolic C2 domains lacking a transmembrane domain. The DOC2 family has been implicated in SV trafficking [119–122], but they appear to represent a novel adaptation to the process found only in vertebrates.

The domain structure of the SYT family is highly conserved across all isoforms, with a single-pass transmembrane domain, a variable linker and two tandem cytosolic C2 domains, termed C2A and C2B [98–100, 123, 124]. C2 domains are found in a wide array of proteins and often function as Ca^2+^-dependent lipid binding modules. They represent one of several Ca^2+^ binding domains found among proteins, with EF hands representing another prominent motif that translates intracellular Ca^2+^ rises into downstream effector responses [125–128]. Within the protein kinase C (PKC) family, the C2 domain serves to bring the kinase to the plasma membrane in response to internal Ca^2+^ elevation through its lipid-binding properties. Such an evolutionarily conserved role can be postulated for some SYT family members, which are tethered to intracellular membrane organelles like SVs via their transmembrane domains. This would allow the C2 domains of SYTs to bridge distinct membrane compartments and bring two lipid bilayers in close proximity for potential fusion or lipid mixing. Although C2 domains are largely considered Ca^2+^-dependent lipid binding modules, they also mediate a host of Ca^2+^-independent interactions. As noted above, multiple SYT family members lack the required aspartate residues that coordinate Ca^2+^ binding, suggesting these isoforms use the C2 domain as a protein–protein interaction module instead. This is also the case for SYTs that display Ca^2+^-dependent lipid binding, as other regions of the C2 domain interact with multiple effector proteins, including the SNARE complex [38, 129]. In addition, a polybasic motif on the surface of the C2 domains of some SYT proteins can interact with lipids in a Ca^2+^-independent manner to facilitate vesicle docking [56, 98, 130–132].

Beyond the role of specific isoforms in SV fusion, SYT family members also function as Ca^2+^ sensors for DCV fusion that mediates release of neuropeptides and neuromodulators [108, 133–142]. Though Ca^2+^ regulation of SV and DCV release by SYTs in presynaptic terminals represent the most-well characterized role, there is also evidence they function in postsynaptic vesicle trafficking [97, 104, 105, 143–148]. Postsynaptic Ca^2+^ influx is required to regulate membrane trafficking to support retrograde signaling and postsynaptic neurotransmitter receptor cycling. Family members of the SYT4/SYT11 and SYT3 subgroups, along with SYT1 and SYT7, have been implicated in these postsynaptic processes. Finally, the group of SYT proteins with degenerate Ca^2+^ binding sites within their C2 domains are likely to participate in distinct Ca^2+^-independent membrane trafficking steps [98–100]. In general, little is known about these more obscure SYT isoforms.

## Synaptotagmin 1 functions as the major Ca^2+^ sensor for triggering synchronous SV fusion

SYT1 is the best characterized member of the SYT family. It is found on SVs and functions as the primary Ca^2+^ sensor for activating fast synchronous fusion in all species examined to date. In mammals, SYT1 is the most highly expressed member of the SYT subgroup found on SVs and serves as the sole Ca^2+^ sensor for synchronous fusion for most neurons in the CNS. SYT2 and SYT9 share largely redundant roles with SYT1 in regulating SV fusion for a smaller population of CNS neurons and most PNS neurons [60, 101]. In Drosophila, a single gene encodes SYT1 and its loss disrupts evoked release as well [49, 51, 57, 63, 75, 149–156]. Although the role of SYT1 as a Ca^2+^ sensor for fusion is widely accepted, the protein also functions in additional steps of the SV cycle, including docking, priming, and endocytosis [57, 150, 155, 157]. We focus our discussion primarily on SYT1 studies performed in Drosophila, as multiple reviews describing mammalian SYT1 are available [40, 44, 70, 86, 158–163].

The multi-functional nature of SYT1 has made it challenging to separate its role for specific steps in SV trafficking, as null mutations disrupt all its properties. This has been partially addressed in Drosophila using point mutants altering a single amino acid, or by rescuing null mutants with SYT1 transgenes containing mutations in one or several residues [49, 51, 57, 62, 75, 131, 150, 152, 156, 164–168]. Although these approaches have improved resolution, several functions of SYT1 are likely to require similar interactions and are difficult to separate. A second issue is that some point mutants have dominant-negative activity and disrupt release more than the complete absence of SYT1, confounding interpretations for these alleles. This has been particularly problematic for C2B domain Ca^2+^ binding mutants that act in a dominant-negative manner [49, 57, 62, 75, 156]. A final issue in interpreting defects in *Syt1* mutants has been the predominant use of the 3rd instar NMJ preparation for analysis. Although this synaptic connection is excellent for physiological studies, it requires animals to progress through earlier larval stages that last for days where compensation through homeostatic mechanisms could mask some phenotypes. In addition, reduced activity in *Syt1* mutants may have consequences on synaptic development. A few studies have used the more difficult embryonic NMJ preparation where developmental defects and compensation are less likely to occur [57, 75, 151]. Acute inactivation approaches have also been used to avoid these issues, though their specificity is somewhat unclear [157, 169].

**Fig. 4:**
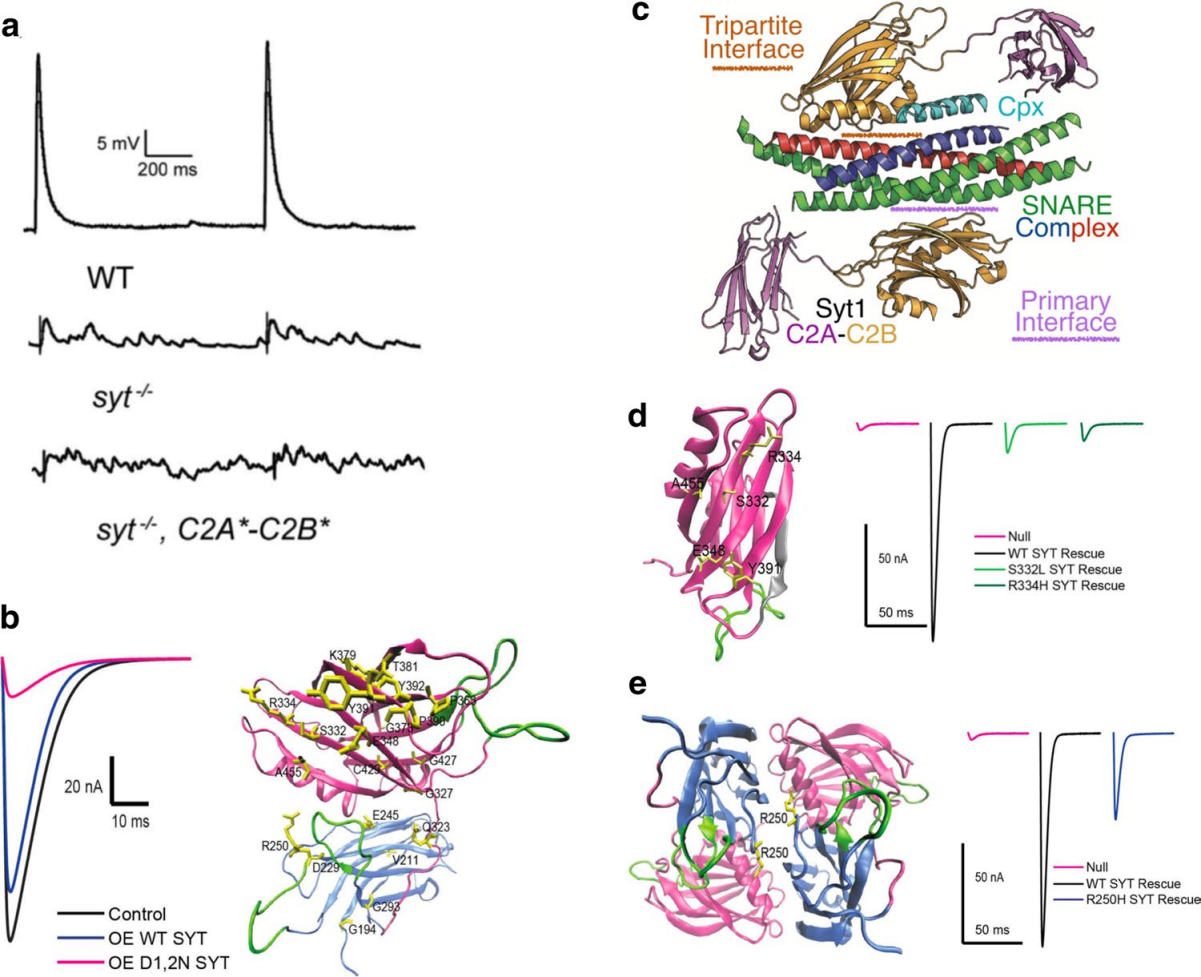
Function of SYT1 in SV fusion. **a** *Syt1* null mutants reduce synchronous fusion and enhance asynchronous release and mini frequency. Rescue with a *Syt1* transgene with defective Ca^2+^ binding to C2A(*) and C2B(*) fails to support synchronous fusion and causes higher rates of spontaneous release. **b** Overexpression of a C2B Ca^2+^ binding mutant (D1,2N) suppresses release compared to overexpression of wildtype SYT1. Twenty essential residues mapping to C2A (blue) or C2B (magenta) were identified in an intragenic suppressor screen that blocked the dominant-negative effects. **c.** Structure of the primary and tripartite interface of the SYT1/SNARE/CPX complex. **d** Location of mutations disrupting the primary SNARE interface on the SYT1 C2B domain. The polybasic stretch is shown in grey and localizes to the opposite C2B surface. SNARE-binding mutations fail to rescue release defects in *Syt1* null mutants (right panel). **e** Location of the R250H mutation at the SYT1 dimer interface that disrupts oligomerization. This mutation reduces SV release (right panel), though not as severely as SNARE-binding mutants. **a** Modified from [49], **b**, **d**, and **e** modified from [62], and **c** modified from [38]

The initial analysis of Drosophila *Syt1* mutants revealed multiple defects in SV release at larval NMJs. The first key observation was that evoked release was dramatically reduced while spontaneous mini frequency was elevated (Fig. 4a) [63, 154]. This result indicated SYT1 played distinct roles in SV release by promoting evoked fusion and clamping Ca^2+^-independent spontaneous release. Although reductions in evoked release can be secondary to many potential defects in the SV cycle, the finding that the Ca^2+^ dependence of release was altered in some *Syt1* hypomorphic mutants provided support for the protein acting as a Ca^2+^ trigger for fusion [51]. Subsequent work in mice identified specific point mutants in SYT1 that shifted the Ca^2+^ sensitivity of release, further pointing towards a release defect tied to Ca^2+^ sensing [170]. Following the initial characterization, additional studies of Drosophila *Syt1* mutants indicated its role in evoked release was twofold. Loss of SYT1 caused a dramatic reduction in fast synchronous release with enhanced release through the slower asynchronous fusion pathway at both embryonic and larval NMJs [57, 75, 151]. This work confirmed SYT1 was not the sole Ca^2+^ sensor for SV fusion, consistent with earlier studies on null mutants [102]. A two Ca^2+^ sensor model for SV exocytosis emerged in the field (Fig. 1c), with SYT1 driving the majority of rapid synchronous release and an unknown Ca^2+^ sensor activating asynchronous fusion. Candidates for the asynchronous Ca^2+^ sensor included other SYT family members, with SYT4 and SYT7 being the only attractive isoforms in Drosophila given their broad expression throughout the nervous system [100]. However, electrophysiological studies of *Syt1*/*Syt4* and *Syt1*/*Syt7* double mutants found these animals retain asynchronous release [153, 171], indicating Ca^2+^-sensitive mechanisms outside of the SYT family contribute to the slower release pathway at Drosophila synapses.

Initial models for SYT1 function in autaptic neuronal cultures from *Syt1* mutant mice suggested loss of the protein resulted in shifts in the time course of fusion without altering the actual number of SVs released. This view was not consistent with the dramatic reduction in the total number of SVs released in Drosophila *Syt1* mutants. Subsequent studies in mice confirmed observations from Drosophila that the total number of SVs released was dramatically reduced in the absence of SYT1 [53, 60, 64, 84, 101]. Quantal imaging of SV release at individual AZs in Drosophila *Syt1* mutants further demonstrated a profound reduction in release probability [22], confirming loss of SYT1 reduces synchronous SV fusion and dramatically decreases the total number of SVs released overall. The dual roles of SYT1 in activating synchronous release and suppressing asynchronous fusion were genetically separated and mapped to distinct C2 domains [75]. These observations supported a model whereby SYT1 actively inhibited the asynchronous pathway, versus the alternative where SVs normally destined for synchronous release were simply being released asynchronously. Together, these data suggested some similarities between SYT1 and rapidly inactivating ion channels that open to allow ion flow and undergo a second conformational change that inactivates the channel. For SYT1, Ca^2+^ binding triggers a conformational state or set of interactions that increase the likelihood of a SV undergoing fusion within several milliseconds. Shortly after, SYT1 undergoes another conformational change, potentially triggered by Ca^2+^ unbinding, that actively inhibits SV release and reduces the probability of any slower asynchronous fusion events. This dual activity of SYT1 helps ensure SVs are released within precise temporal windows that are needed for rapid neuronal computations.

Beyond a role in regulating fusion, a number of studies also identified defects in SV docking, priming and endocytosis, indicating SYT1 acts at multiple steps to regulate SV cycling in Drosophila [57, 150, 155, 157]. Although *Syt1* mutants have defects in docking and endocytosis, the protein is not essential for either pathway as these processes continue with reduced efficacy in the absence of the protein. Interactions between SYT1 and clathrin adapter proteins provide candidate mechanisms for its role in facilitating endocytosis [150, 172–179]. In contrast, how SYT1 supports SV docking is less clear. Ca^2+^-independent membrane interactions mediated by a polybasic amino acid stretch in the two C2 domains, together with its SNARE-binding properties, represent candidate mechanisms. Overall, initial studies of SYT1 provided models for how it regulates SV cycling. Loss of SYT1 did not eliminate spontaneous or asynchronous release and had no obvious effects on spontaneous SV fusion kinetics. As such, SYT1 does not appear to be essential for fusion or dynamics of the SV fusion pore itself, but acts as a Ca^2+^-dependent trigger to increase the likelihood SNARE-dependent fusion occurs and is limited to a short temporal window. Although the kinetics of single SV fusion events do not appear to be controlled by SYT1, there is evidence the protein can regulate fusion pore kinetics of DCVs and in reconstituted in vitro fusion systems [138, 139, 180–188]. These early studies in Drosophila also highlighted differences between the mechanisms for fast synchronous SV release that require SYT1 versus asynchronous and spontaneous fusion that are negatively regulated by the protein.

## Role of C2 domain Ca^2+^ binding and SNARE interactions for Synaptotagmin 1 function

To directly characterize the role of the C2 domain Ca^2+^ binding loops in Drosophila, a subset of the key aspartate residues in each loop (numbered D1–D5) were mutated to neutralize the negative charge and prevent Ca^2+^ binding. The most common approach was to generate D to N substitutions to create D1,2N and D3,4N mutants in either or both C2 domains and use these transgenes to rescue *Syt1* null mutants. This approach indicated the C2B domain had a critical role in evoked release, with a near complete loss of synchronous SV fusion in C2B D1,2N or C2B D3,4N mutants [49, 75, 156]. Similar transgenes disrupting the C2A domain did not block evoked release, but rather failed to clamp asynchronous fusion [49, 75]. Together with studies in mice, these findings pointed towards Ca^2+^ binding to the C2B domain as the critical trigger for exocytosis, while C2A Ca^2+^ binding appeared less important. However, these results have become more difficult to interpret with the observation that mutations in the C2B Ca^2+^ binding pocket cause dominant-negative phenotypes in Drosophila, mice, and humans [49, 57, 75, 152, 156, 164, 189–191]. Indeed, release in *Syt1* null mutants rescued with C2B D1,2N or C2B D3,4N display less SV fusion that the null mutant itself [75, 156]. This has led to re-evaluation of how these substitutions are altering C2 domain function. One hypothesis is neutralization of the charged residues makes SYT1 more likely to undergo Ca^2+^-independent membrane interactions via the loops that potentially block fusion sites. As such, the preferential role of Ca^2+^ binding to C2B as the sole trigger for SV fusion requires re-evaluation. More recent mutagenesis work using different amino acid substitutions within the C2 domain loops that alter hydrophobicity of residues rather than charge indicate C2A Ca^2+^ binding also has an important role in SYT1 [165, 192–195]. These observations suggest Ca^2+^ binding to both C2 domains is important for the full activity of SYT1 in triggering synchronous SV fusion. Mutations disrupting Ca^2+^ binding to both C2 domains of SYT1 still support SV docking and endocytosis, indicating these functions of the protein are largely Ca^2+^-independent [49], though Ca^2+^ binding may enhance endocytosis rates [176]. Interestingly, C2A–C2B Ca^2+^ binding-defective SYT1 animals have a far greater increase in spontaneous release compared to null mutants (Fig. 4a), indicating restoring docking and endocytosis magnifies the role of SYT1 in clamping spontaneous fusion that is less pronounced in nulls due to reduced SV number [49].

One candidate mechanism for Ca^2+^-dependent membrane binding by SYT1 is based on structural similarity of the C2 domain loops to fusion peptides in viral fusion proteins like hemagglutinin [196]. For viral fusion proteins, conformational changes in the acidic environment of endosomes expose the loops, allowing them to drive fusion between viral and endosomal membranes and release viral content. The fusion loops in SYT1 contain negatively charged aspartate residues nested within them, unlike the loops of viral fusion proteins. In the absence of Ca^2+^, these residues repel C2 domains from interacting with negatively charged phospholipid containing membranes. In the presence of Ca^2+^, the negative charge on these residues are neutralized and the C2 domains engage the lipid bilayer, potentially allowing the loops to function similarly to those of viral fusion proteins.

Beyond the role of Ca^2+^-dependent lipid binding by the C2 domains, several other SYT1 interactions have been implicated in SV release in Drosophila. *Syt1* transgenes containing mutations in the C2B Ca^2+^ binding pocket dominantly disrupt release (Fig. 4b) and cause lethality even in the presence of endogenous SYT1. Taking advantage of this observation, chemical mutagenesis screens have been performed to identify intragenic SYT1 suppressors where randomly generated second-site mutations in the dominant-negative transgene reduce the toxic effect [62]. This approach uncovered 20 essential residues within SYT1 that mapped to distinct areas of the protein (Fig. 4b). The screen identified the five essential C2B domain residues (S332 (S279 in mammalian SYT1), R334 (R281), Y391 (Y338), E348 (E295) and A455 (A402) that form the primary surface interaction site that docks one side of the C2B domain onto the SNARE complex based on the elucidated structure (Fig. 4c) [129]. Generation of several of these mutations into otherwise wildtype *Syt1* transgenes demonstrated they failed to rescue evoked release defects in *Syt1* nulls (Fig. 4d). Disruption of the SYT1–SNARE complex interaction caused a loss of synchronous release, enhanced asynchronous fusion, a reduction in the Ca^2+^ cooperativity curve, and elevated spontaneous release. As such, these SYT1 functions require SNARE complex binding. SV docking and endocytosis defects were not observed, suggesting these roles are independent of SNARE binding or redundant with other SYT1 binding partners.

Beyond SNARE interactions, mutations that disrupt the C2B polybasic stretch on the opposite surface of the C2B SNARE-binding domain were also identified in the screen. In the absence of Ca^2+^, SYT1 binds to PIP2 present in the plasma membrane through a polybasic stretch of amino acids lining this surface of the C2B domain. This interaction is predicted to occur before fusion as part of the docking/priming process, helping to increase membrane penetration of the C2 loops during Ca^2+^ entry by prepositioning SYT1 at fusion sites [44, 56, 130, 132, 165, 197–202]. Similar to prior structure function-studies, disruption of this Ca^2+^-independent lipid binding surface reduced evoked release, but caused far milder phenotypes than loss of Ca^2+^-dependent lipid binding or SNARE interactions [62, 131]. Together, these data suggest a model where SYT1 is prepositioned at the SV-plasma membrane interface in part through its C2B polybasic stretch and more fully by its C2B SNARE-binding interaction. Upon Ca^2+^ entry, the C2A and C2B Ca^2+^ binding loops pivot into the plasma membrane to drive lipid instability and facilitate complete SNARE zippering to activate fusion [44, 56, 203–206]. An alternative model includes two separate pools of SYT1 on the SV, one bound to SNARE complexes to support docking/priming and another pool not associated with SNAREs that mediate Ca^2+^-dependent lipid binding and membrane insertion of the protein.

The role of SYT1 tethering to SVs has also been examined by generating transgenes lacking the transmembrane domain or replacing the transmembrane domain with a myristoylation motif [152]. These transgenes could not support fast synchronous fusion in *Syt1* null mutants, though cytosolic C2 domains enhanced asynchronous release even more than that observed in *Syt1* nulls. These observations suggest transmembrane tethering of SYT1 positions the protein near release sites so its Ca^2+^ binding properties can rapidly drive fusion. An additional study examined the relevance of the individual SYT1 C2 domains for release by generating otherwise wildtype SYT1 proteins with only C2A or C2B alone, double C2A–C2A or C2B–C2B modules or reversed C2B–C2A order [49]. None of these manipulations rescued release defects in *Syt1* null mutants, indicating both C2 domains are uniquely required for release and must be present in the correct sequence. Similar observations have been made for C2 domain swaps between mammalian SYT1 and SYT7, where chimeric proteins failed to rescue *Syt1* mutant phenotypes in mice [207]. Although C2 domains may have a generalized function, they have uniquely evolved within each SYT family member, with the specific C2A and C2B domains serving distinct roles.

## Open questions on the role of Synaptotagmin 1 in SV trafficking and fusion

It is widely accepted that the central role of SYT1 is centered around Ca^2+^-dependent lipid binding, but questions about additional interactions and their role in fusion are still being debated. For example, the precise role of SNARE binding is less clear. Studies in Drosophila provide strong in vivo evidence this interaction is required for SYT1 to promote synchronous fusion and suppress asynchronous release, but it is unknown when the interaction occurs during the SV cycle. Part of this confusion is tied to the lack of a precise understanding of the structure of the primed SNARE complex before fusion in vivo. Are SNARE complexes partially zippered before fusion as most models suggest, and can SYT1 bind to partially assembled SNAREs in this state? If so, this would place the interaction at a pre-fusion point to position SYT1 for future membrane insertion and/or regulate SNARE zippering. The interaction could also contribute to multimerization of SNARE complexes through the oligomerization properties of SYT1. Alternatively, the interaction might require fully assembled *trans*-SNARE complexes that are predicted to only form during fusion. This would place the interaction during the fusion process itself or after fusion has been completed. Although early biochemical work suggested SYT1 binding to individual t-SNAREs and the t-SNARE complex was Ca^2+^-dependent, current data suggest the primary binding mode is Ca^2+^-independent. As such, SNARE binding could act before, during or after Ca^2+^ entry.

Several other binding modes between SYT1 and the SNARE complex have also been described beyond the primary interface on the C2B surface that has genetic support in Drosophila. One model based on X-ray crystal structure suggests formation of an alpha helix partially contributed by a piece of the SYT1 C2B domain and part of the SNARE-binding protein CPX (Fig. 4c) [38]. This “tripartite” interaction mode brings in another key component for fusion regulation in CPX, but no genetic data has implicated this region of SYT1 in Drosophila SV trafficking. However, studies in Drosophila indicate the activity of SYT1 and CPX are indeed intimately tied together in controlling SV release dynamics [37]. *Cpx* null mutants share similar phenotypes with milder *Syt1* loss-of-function alleles. Both mutants have decreased synchronous release, enhanced asynchronous fusion and elevated rates of spontaneous release [37, 41, 208–210]. Although SYT1 is the primary candidate for a fusion clamp to prevent spontaneous release in mammals, that role is mediated more prominently by CPX at invertebrate synapses. *Syt1* null mutants and animals with mutated C2A/C2B Ca^2+^ binding sites display a two–tenfold increase in mini frequency compared to controls [37, 49, 51], while *Cpx* null mutants have a far greater increase that can exceed 100-fold [36, 37, 41, 95, 208–213]. As such, both proteins negatively regulate spontaneous release by clamping fusion, but the balance of their effects have shifted during evolution. SYT1 and CPX also display genetic interactions in double mutants or following co-overexpression, suggesting CPX is likely to exert many of its effects by regulating the timing and activity of SYT1 during SV priming and fusion in Drosophila [37]. An attractive hypothesis involves CPX binding to the partially assembled SNARE complex to regulate when and how SYT1 interacts, allowing additional spatial and temporal control of SYT1-SNARE interactions during priming and fusion.

Another question remaining in the field is how many SNARE complexes and SYT proteins are required for fusion in vivo. Most estimates suggest 3–5 SNARE complexes drive evoked fusion, while the assembly of only a single SNARE complex might be sufficient to trigger spontaneous release [214–217]. Increasing the number of assembling SNARE complexes has been shown to increase release probability for SV fusion in vivo [31]. In terms of the number of SYTs required, estimates suggest ~ 15 SYT proteins are found on a single SV [218]. Do SYTs have to bind every SNARE complex at the fusion site or is binding to only one sufficient? Beyond the number of SYTs that bind SNAREs, how many SYTs are required overall to trigger an evoked fusion event and inhibit asynchronous and spontaneous release? If more than one SYT is required, do they need to form an oligomeric complex, or can they act independently? Do the number of SYT proteins activated during Ca^2+^ entry contribute to the steep Ca^2+^ cooperativity curve for SV fusion, or is that effect mediated through other mechanisms? Do individual SYTs bind SNAREs, while others drive Ca^2+^-dependent membrane insertion? What actual changes in the lipid structure of the presynaptic membrane occur following penetration of the SYT1 C2 domains that activates fusion? How specifically do SYT1 proteins prevent asynchronous and spontaneous release; are these shared or distinct mechanisms from its positive role in activating synchronous release? Defining the timing and precise role for each of SYT1’s interactions in the various routes of SV trafficking and fusion will require future studies with additional temporal and spatial resolution.

Additional questions in the field center on the role of oligomerization in SYT1 function. Although SYT1 forms oligomers in vitro [49–51, 219–224], the importance, stability, and timing of SYT1 oligomerization for SV release in vivo is unclear. Distinct SYT1 oligomerization states may also exist, as multimerization can occur via Ca^2+^-dependent and Ca^2+^-independent mechanisms and through interactions mediated by the linker domain, residues on the C2A surface or via the C2B polybasic region [49, 82, 177, 219, 221–227]. In the intragenic suppressor screen described above, an oligomerization-defective C2A mutant (R250H) was identified [62]. This mutant reduced evoked release but did cause disruptions to the synchronicity of fusion (Fig. 4e). As such, the R250H mutant suggests a potential role for oligomerization in enhancing the number of SVs that fuse, with release timing and suppression of asynchronous fusion independent of oligomerization. Although oligomerization is defective in R250H, this mutation also disrupts C2A–C2B intradomain interactions within individual SYT1 monomers, suggesting either interaction could contribute to defects observed in this mutant. It is also possible that other oligomerization states function independently of the C2A R250 residue to drive SYT1 activity. Defining the role of SYT1 multimers in SV trafficking requires future studies in the field.

## Is Synaptotagmin 7 the Ca^2+^ sensor for asynchronous release?

The discovery that SYT1 mediates synchronous release but does not abolish asynchronous fusion raised the possibility that other members of the SYT family might function as the asynchronous Ca^2+^ sensor. SYT7 emerged as a promising candidate due to its higher affinity for Ca^2+^, strong lipid binding properties, slower dissociation from membranes, and widespread expression within the nervous system [228–233]. These properties could potentially allow SYT7 to activate SV fusion farther away from AZs and after the initial rise in Ca^2+^ influx (Fig. 1c). This would provide an attractive solution to the Ca^2+^ sensing problem for SV fusion: SYT1 functioning as the synchronous sensor and its homolog SYT7 acting as the asynchronous sensor. In contrast to the work on SYT1 in Drosophila, which generated many of the initial insights into in vivo mechanisms, studies of SYT7 have been primarily done in mice. As such, we discuss these data sets in more detail and compare them to recent studies on the Drosophila SYT7 homolog.

**Fig. 5:**
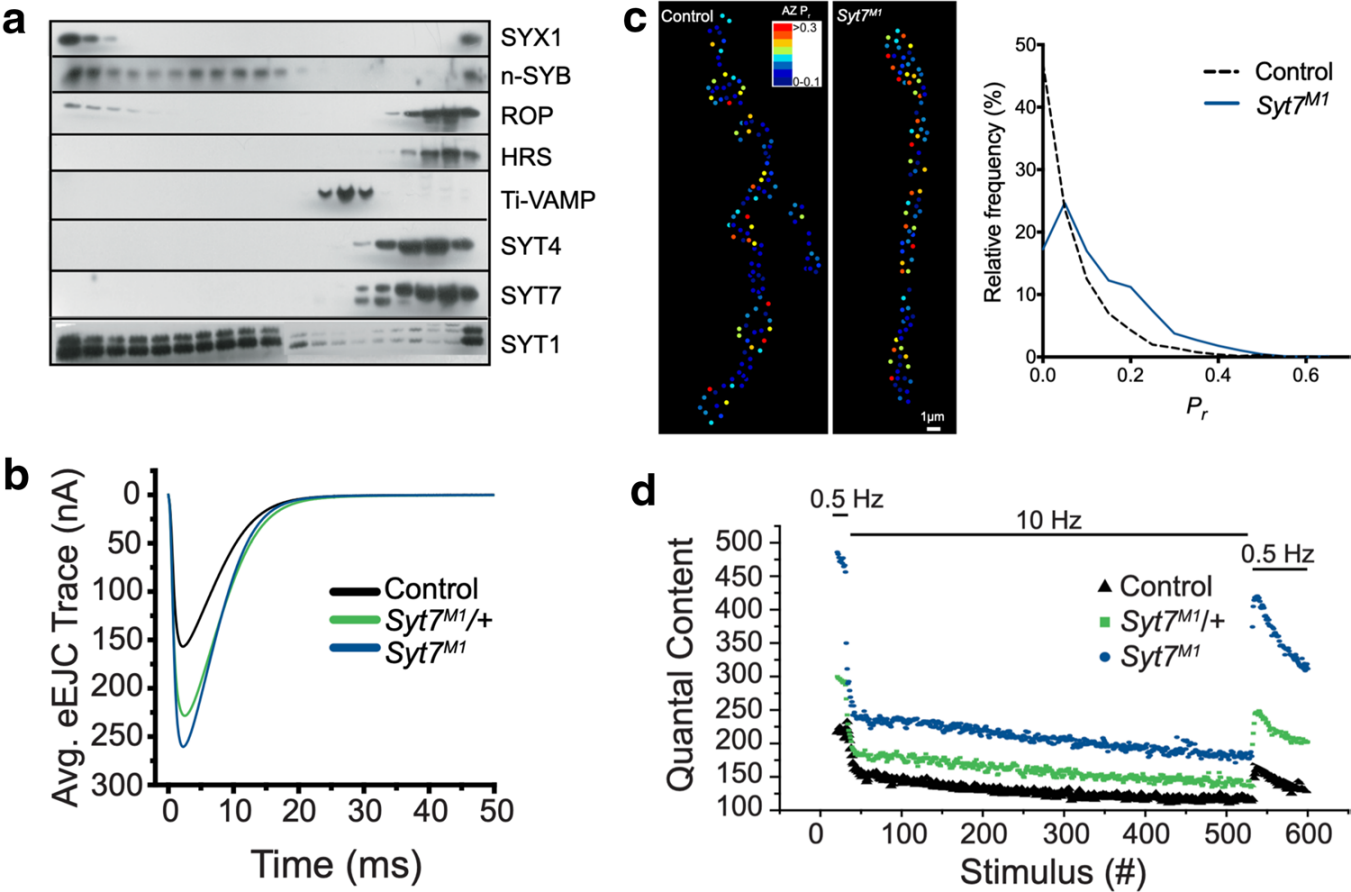
Enhanced release and SV replenishment in *Syt7* mutants. **a** Western analysis of Drosophila brain extracts separated on a 10–30% sucrose gradient. SYX1 labels the plasma membrane (left-most fraction) with SVs (n-SYB/SYT1) in intermediate fractions. SYT7 and SYT4 fractionate with other internal membrane compartments to distinct regions of the gradient. **b** Evoked release is increased in *Syt7* null mutants (*Syt7*^*M1*^) and *Syt7* heterozygotes. **c** Optical mapping of release probability at larval NMJs demonstrate *Syt7* mutant AZs are shifted to higher *P*_*r*_ compared to controls. **d** *Syt7* mutants undergo rapid depression during a stimulation train and recover their releasable SV pool more quickly than controls. Heterozygotes show an intermediate phenotype. Panel **a** modified from [100] and panels **b–d** modified from [82]

Drosophila SYT7 was not identified until the genome was sequenced in 2000 [99, 234, 235]. *Syt7* resides on the small 4th chromosome in a genomic region that had been poorly characterized in the early 2000s and that lacked genetic toolkits to easily generate mutations. As such, the protein remained uncharacterized for many years. The first efforts to examine Drosophila SYT7 function used RNAi knockdown, RNA in situ hybridization and immunocytochemical studies with antibodies raised against the protein [100, 153]. These approaches demonstrated *Syt7* mRNA was abundantly expressed in most neurons at levels similar to *Syt1* and *Syt4*. Recent RNA profiling also indicates *Syt7* mRNA is abundant in neurons (Fig. 3). Initial RNAi approaches that attempted to reduce the levels of SYT7 failed to show a synaptic phenotype [153]. SYT7 did not co-localize with SYT1 on SVs or enrich on synaptic plasma membranes with the t-SNARE Syntaxin on brain extracts fractionated from sucrose gradients (Fig. 5a) [100]. Similarly, CRISPR tagging of endogenous SYT7 with GFP or RFP showed the protein was present in presynaptic terminals but not localized to SVs [82]. Unlike an early study of mammalian SYT7 which identified the protein at the AZ plasma membrane [232], no enrichment of SYT7 at Drosophila AZs was observed. Subsequent work in mammals have reported SYT7 localization in multiple compartments, including the plasma membrane, DCVs, lysosomes, endosomes and other internal membrane compartments [100, 232, 236–243]. These diverse findings have made it unclear as to which membrane compartment contains SYT7, or if the protein is present in multiple locations that could participate in trafficking of distinct organelles.

Further characterization of CRISPR-tagged SYT7 in Drosophila found the protein was expressed in a highly interconnected tubular membrane compartment in presynaptic boutons that made partial contact with numerous organelles, including endosomes, lysosomes and the plasma membrane [82]. Although SYT7 did not fully co-localize with any known compartmental marker, the protein was enriched within the peri-AZ (Fig. 3d, e), a synaptic domain surrounding AZs that contain proteins involved in endocytosis and protein sorting [82, 244–250]. Recent characterization of SYT7 localization using STORM in mouse hippocampal neurons also found the protein at the periphery of AZs [251], though it is unclear if this represents localization to the plasma membrane versus an internal SYT7 compartment. If SYT7 functions as the asynchronous Ca^2+^ sensor that mediates SV fusion with the plasma membrane, it should reside on SVs or at the AZ presynaptic membrane. SYT7 does not appear to be enriched on either of these compartments in Drosophila. Given the primary localization of SYT7 within the peri-AZ, functions beyond directly mediating fusion of SVs with the presynaptic plasma membrane must be considered.

Initial studies using Drosophila SYT7 RNAi knockdown and mouse *Syt7* mutants did not suggest a role for SYT7 in asynchronous release [153, 252]. The first observation indicating SYT7 might function in this slower release pathway was based on morpholino knockdowns of zebrafish SYT7 that eliminated asynchronous release at NMJs [78]. This finding triggered a wave of new studies to re-evaluate SYT7’s role. Additional characterization of mammalian SYT7 using siRNA knockdown suggested the protein was indeed responsible for asynchronous release in excitatory and inhibitory neurons [84]. These phenotypes were found by knocking down SYT7 in SYT1 deficient neurons with enhanced asynchronous release, or in wildtype neurons during high frequency stimulation, making it easier to detect changes in the asynchronous pathway. Subsequent work in mouse *Syt7* mutants supported a role for SYT7 as the asynchronous Ca^2+^ sensor [80, 83, 253–256]. Beyond asynchronous release, some studies found manipulations of SYT7 also disrupted facilitation, a form of short-term plasticity where SV release is enhanced during closely spaced stimuli [80, 81, 257]. These data supported a model that SYT7 acts as the Ca^2+^ sensor for both asynchronous release and facilitation in a subset of mammalian neurons.

Although much of the literature converged on the two Ca^2+^ sensor model for SV fusion, the role of SYT7 in asynchronous release remains controversial. Asynchronous release at several mammalian CNS synapses is anti-correlated with the levels of the synchronous Ca^2+^ sensors SYT1 and SYT2 and does not correlate with SYT7 levels [58]. In addition, another study found no defects in asynchronous release in mouse *Syt7* mutants [76]. Mammalian SYT7 has also been suggested to play distinct roles in the SV cycle, including driving SV replenishment during high frequency stimulation [76], refilling of the readily releasable pool (RRP) [258], and targeting of SVs to specific endocytic pathways [77]. Such defects could potentially contribute to decreases in asynchronous fusion due to fewer SVs available for this phase of release. In addition to these roles, mammalian SYT7 regulates fusion of lysosomes with the plasma membrane in non-neuronal cells and DCV release in certain neuronal populations [83, 133, 134, 237, 240–243, 256, 259–263].

The discrepancies in mammalian studies make it difficult to determine if SYT7 has a conserved primary role at synapses, or if the protein has distinct functions in subpopulations of neurons. To examine conserved roles for SYT7 in Drosophila, CRISPR mutants in the locus were recently generated [82]. Consistent with mice lacking SYT7, Drosophila lacking the protein are viable and fertile, indicating SYT7 is not required for synaptic transmission or non-neuronal fusion events essential for survival. However, *Syt7* mutants displayed numerous defects at NMJ synapses in 3rd instar larvae, including a large increase in evoked SV release, a larger RRP vesicle pool, and faster SV replenishment after strong stimulation (Fig. 5). Consistent with increased release, mutant terminals displayed depression during stimulation trains compared to controls that facilitated in the same extracellular [Ca^2+^]. Lowering extracellular Ca^2+^ to reduce release probability in *Syt7* mutants restored facilitation, indicating the process itself is intact at synapses without SYT7. These release defects were dosage-sensitive, with *Syt7/*+ heterozygotes showing intermediate increases in SV fusion (Fig. 5b) and SYT7 overexpression suppressing evoked and spontaneous release. Similar dosage effects appear to be present at some mammalian synapses [264]. Optical imaging of evoked release in Drosophila *Syt7* mutants demonstrated a higher release probability at single AZs compared to controls (Fig. 5c) [82]. The enhancement in evoked release resulted in less asynchronous fusion and facilitation compared to controls, as observed in mammals. However, increased release probability leads to faster SV depletion and reduces the number of SVs available for fusion via these pathways. These data suggest a model where the levels of SYT7 act as a dial to decrease or increase the amount of release to enable or mask facilitation mediated by an independent Ca^2+^ sensor, suggesting modulation of *Syt7* transcription or SYT7 protein levels could play a central role in short-term plasticity.

To conclusively test if asynchronous release and facilitation requires SYT7, electrophysiological recordings were performed in *Syt1*, *Syt7* double mutants [82]. In animals lacking both proteins, an enhancement of both evoked and spontaneous release compared to *Syt1* null mutants alone was observed. In addition, double mutants still displayed synaptic facilitation. These results indicate SYT7 negatively regulates release probability for SVs destined for release through both the spontaneous and evoked pathways in the absence or presence of SYT1. Since evoked release remains in animals lacking both SYT isoforms, the popular two Ca^2+^ sensor model of SYT1 acting as the synchronous sensor and SYT7 as the sole asynchronous sensor is not applicable for Drosophila synapses. Similarly, the lack of SYT7 accumulation on SVs or at the AZ plasma membrane make it unlikely Ca^2+^ binding to SYT7 could directly trigger SV fusion since the protein is not physically present at release sites. Given similarity in release kinetics and Ca^2+^ sensitivity for asynchronous fusion between Drosophila and mammalian synapses [57, 75], it is perplexing that SYT7 could take over this role at mammalian synapses while a distinct Ca^2+^ sensor mediates asynchronous release in Drosophila. An alternative model is that SYT7 controls the number of SVs available for asynchronous release, but a distinct Ca^2+^-sensitive mechanism directly mediates fusion through this slower pathway.

Although more work is required to understand how SYT7 negatively regulates SV trafficking in Drosophila, some similarities and differences between invertebrate and mammalian systems can be noted. Lack of SYT7 results in defects in asynchronous release and facilitation, though the hypothesized mechanisms are very different (Ca^2+^ sensor for asynchronous fusion in mammals, negative regulator of release probability in Drosophila). To date, increases in SV release probability have not been reported in mammals. Both proteins also negatively regulate spontaneous release, as mini frequency is reduced when SYT7 is overexpressed in either mice or Drosophila [82, 84]. Similarly, spontaneous frequency is elevated in *Syt1*/*Syt7* double mutants in both species [82, 84, 257]. This finding has been interpreted as SYT1 and SYT7 having redundant roles in clamping spontaneous fusion in mammals. In contrast, the Drosophila model argues more SVs are available for release that have to be clamped by SYT1 and/or CPX. SYT7 also regulates the size and recovery of SV pools after stimulation in Drosophila, as *Syt7* mutants have a larger pool of releasable SVs and a faster refilling rate after stimulation (Fig. 5d). The enhanced replenishment of the RRP is still observed in the absence of endocytosis, suggesting it involves changes in movement between existing SV pools [82]. SYT7 overexpression causes the opposite phenotype, reducing the pool of fusogenic SVs and delaying replenishment. Several mammalian studies have identified a role for SYT7 in SV replenishment [76, 79, 191, 258], but the protein promotes refilling in contrast to Drosophila SYT7. One study identified a redundant role for mammalian SYT1 and SYT7 in maintaining the RRP [191], while SYT1 alone acts to positively regulate the RRP in Drosophila [57]. A few studies suggest STY7 controls SV endocytosis in mammals [77, 265], but steady-state endocytosis rates are not altered in Drosophila *Syt7* mutants. Rather, SYT7 regulates how many and how rapidly SVs move between distinct pre-existing pools at Drosophila synapses. Although SYT7 is highly conserved at the sequence level across evolution, its function appears to have diverged more than SYT1. Given conflicting reports on mammalian SYT7 function, some of species differences may also reflect differential expression levels between SYT1 and SYT7 across diverse neuronal populations [58].

Although trafficking defects have now been characterized in Drosophila *Syt7* mutants, how the protein negatively regulates SV release and replenishment from its peri-AZ location is unknown. EM analysis of *Syt7* mutant NMJs did not reveal increased SV number or differences in SV distribution around AZs [82], suggesting the total SV pool is unaltered. These findings indicate loss of SYT7 alters the distribution and movement between existing SV pools that give rise to these phenotypes. Since SYT7 is not found on SVs or at AZs, it likely regulates SV release through an indirect mechanism. SYT7′s location within the peri-AZ positions it to interact with multiple endosomal and recycling compartments that could negatively impact SV trafficking. One model is that SYT7 hetero-oligomerizes with SYT1 to reduce fusogenicity of a SV pool or restrict SVs from entering the releasable pool. Although hetero-oligomerization between distinct SYT family members occurs in vitro [177, 225, 226, 266], the in vivo significance is unclear since most SYT proteins reside in distinct subcellular compartments. Given loss of SYT7 enhances release in the absence of SYT1, this mechanism seems unlikely to be the dominant pathway for SYT7 activity. Another model is that Ca^2+^ binding to SYT7 acts as a sponge to reduce fusion by limiting intracellular Ca^2+^ availability for SYT1 given the steep Ca^2+^ dependence of SV release. SYT7 might also regulate Ca^2+^ influx or extrusion pathways. The lack of SYT7 enrichment at fusion sites and the preservation of enhanced release in *Syt1/Syt7* double mutants argues against these mechanisms. In addition, quantitative imaging of presynaptic Ca^2+^ levels with Fluo-4 AM at *Syt7* mutant NMJs revealed a mild reduction in evoked Ca^2+^ influx rather than an elevation as predicted by this model [82]. Another potential mechanism would involve SVs being more efficiently recruited from the reserve pool (RP) in the absence of SYT7. The SV protein Synapsin helps maintain the RP by tethering SVs to actin filaments [267–279] and is known to interact with other peri-AZ proteins to regulate SV cycling [244, 280]. As such, SYT7 might modulate a Synapsin-dependent process that controls SV availability. Characterization of *Synapsin/Syt7* double mutants could determine if SYT7 normally limits liberation of SVs from the RP.

SYT7 might also regulate SV distribution by directing sorting of newly generated SVs in a Ca^2+^-dependent manner. Studies of *Cpx* mutants, which have a dramatic increase in mini frequency, revealed segregation between SV recycling pathways following spontaneous versus evoked release [213]. Ca^2+^-independent recycling following spontaneous fusion resulted in a rapid re-accumulation of recycled SVs at AZs in the RRP. In contrast, SVs recycled following evoked release when cytosolic Ca^2+^ is elevated were slower to re-enter the AZ pool. SYT7 might act as the Ca^2+^-triggered recycling switch to redirect SVs to an internal pool and delay their return to the AZ. A final more speculative hypothesis for SYT7 function is that it acts similarly to more distantly related proteins like the ESYT family found at ER-plasma membrane contact sites that mediate lipid exchange between distinct membrane compartments [111, 281–285]. If so, SYT7 might function by bringing two presynaptic membrane compartments together in a Ca^2+^-dependent manner to regulate lipid exchange by phospholipid transporters. SYT7 binds to PIP2 with high affinity [231], and limiting plasma membrane PIP2 levels would be an attractive mechanism given this lipid enhances SV release through interactions with both UNC13 and SYT1 [197, 286–291].

Unlike structure–function analysis of Drosophila SYT1, little is known about SYT7’s required molecular interactions. However, it is unlikely the C2 domains of SYT1 and SYT7 regulate SV trafficking through similar mechanisms. In contrast to SYT1, Ca^2+^ binding to the SYT7 C2A domain appears to play the major role in regulating SV release in mammals [81, 84, 231]. The C2 domains of SYT1 and SYT7 are not interchangeable in vivo as SYT1/SYT7 chimeras cannot rescue *Syt1* mutant phenotypes [207]. One difference between isoforms is the HB helix in the C2B domain that negatively regulates SV fusion in SYT1 is not present in Drosophila or mammalian SYT7 proteins [82, 207]. Another difference maps to the primary SNARE-binding surface on the SYT1 C2B domain. Four of the five essential residues required for this interaction in SYT1 are not conserved in SYT7 [62, 82]. Indeed, SYT7 has amino acid substitutions at two of these residues that precisely match previously isolated *Syt1* mutants that block SNARE binding and abolish SYT1 function in SV release [62]. These observations indicate SYT7 does not interact with the SNARE complex or does so through a distinct mechanism from SYT1. Beyond these changes, numerous other nonsynonymous amino acids within the C2 domains are conserved only in SYT1 or SYT7 family members, suggesting extensive divergence during evolution from the last common SYT ancestor. Together, these data indicate SYT1 and SYT7 regulate SV trafficking through distinct mechanisms and from separate membrane compartments. More studies are required to determine mechanistically how SYT7 contributes to SV cycling in both Drosophila and mammals, as well as to determine why asynchronous release defects are found in *Syt7* mutants.

## Synaptotagmin 4 regulates retrograde signaling and is transferred between synaptic compartments by exosomes

The mammalian SYT4 protein was identified in searches for novel SYT genes prior to the advent of genome sequencing [292, 293]. Early studies found SYT4 expressed throughout the mouse brain, but revealed the protein lacked Ca^2+^-dependent lipid binding in vitro. Another family member, SYT11, was also identified that failed to bind Ca^2+^ [294]. The failure to bind Ca^2+^ mapped to an aspartate to serine substitution in a key Ca^2+^ binding aspartate residue (D3) in the C2A domain of SYT4 and SYT11. The mammalian *Syt4* gene was independently identified as an activity-regulated intermediate early gene induced by seizure activity [295]. These two initial observations led to a model that activity-dependent upregulation of a Ca^2+^-insensitive SYT isoform might have a neuroprotective role [296]. By oligomerizing with SYT1 and reducing the ability of the heterooligomer to bind Ca^2+^, SYT4 was hypothesized to reduce SV release following excessive neuronal hyperactivity. This model assumed SYT4 was a resident SV protein like SYT1.

Drosophila encodes a single SYT4 family member and initial studies suggested SYT4 overexpression could reduce SV release [177], consistent with mammalian models for its function. Another early study hypothesized a different role where SYT4 could substitute for SYT1 function in SV release in Drosophila [297]. Both of these hypotheses turned out to be wrong when subsequent work found SYT4 was not a SV protein [100]. Antibodies specific to SYT4 revealed a prominent localization of the protein in puncta within the postsynaptic compartment of muscles at the Drosophila NMJ (Fig. 3c) [97, 100, 104]. This localization suggested SYT4 might instead regulate membrane trafficking within postsynaptic compartments. Drosophila SYT4 was also found to bind Ca^2+^ unlike its mammalian orthologs [97, 104, 298]. Ca^2+^-regulated membrane trafficking is known to occur within postsynaptic compartments and can use similar vesicular trafficking components to those found in presynaptic terminals [100, 144, 299–302]. In Drosophila, retrograde signaling mediated through the release of multiple secreted factors is essential for development and plasticity of the NMJ where it links neuronal activity and synaptic growth regulation [14, 22, 97, 105, 303–308]. To determine if SYT4 regulates postsynaptic vesicular trafficking similar to the role of SYT1 in presynaptic terminals, *Syt4* null mutants were generated in Drosophila [104]. Animals lacking SYT4 were viable and fertile, indicating the protein was not essential for viability. However, electrophysiological and imaging studies supported a role for SYT4 as a postsynaptic Ca^2+^ sensor for retrograde signaling at NMJs [95, 97, 104, 105, 305].

**Fig. 6:**
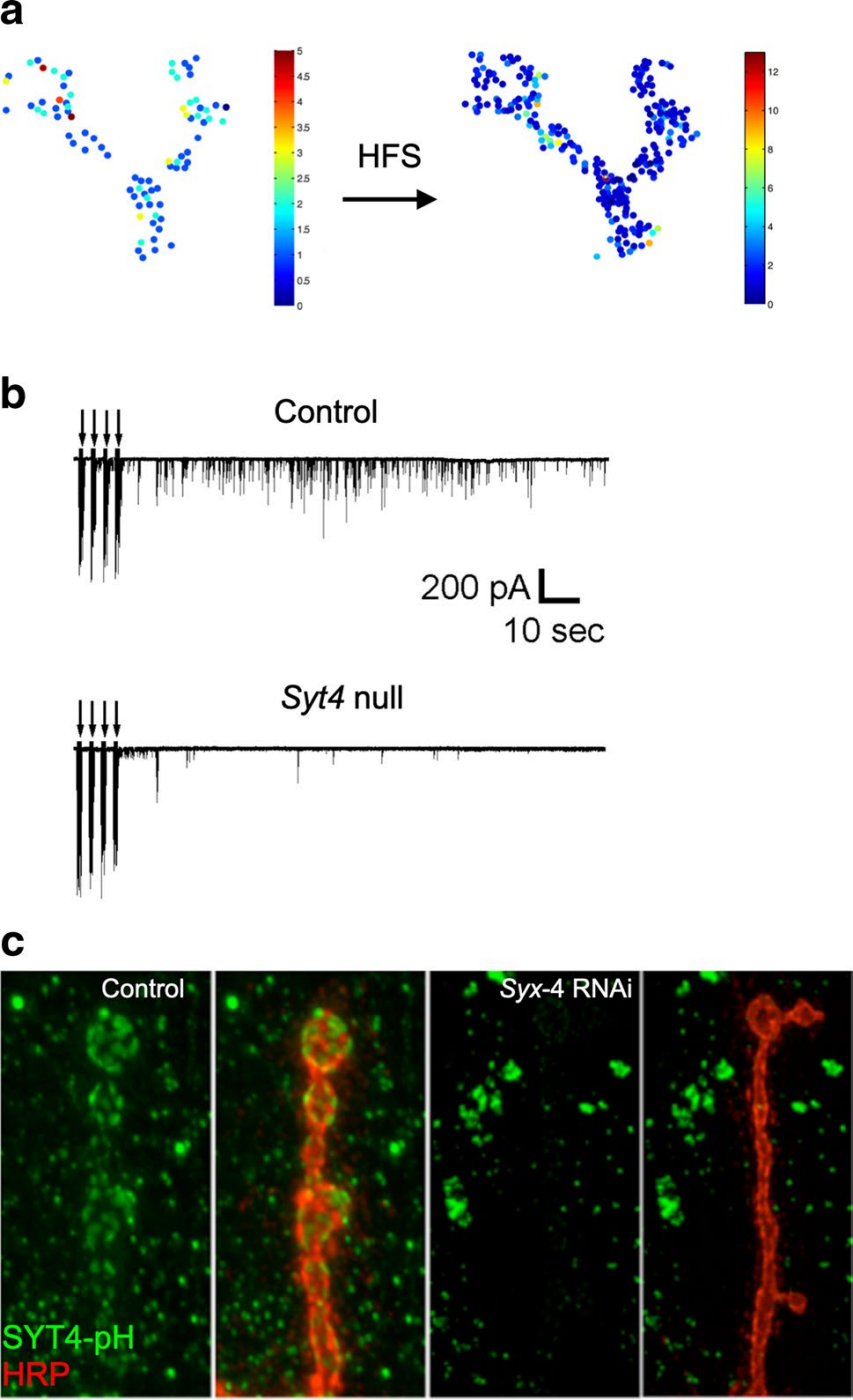
SYT4 controls retrograde signaling at Drosophila NMJs. **a** Drosophila NMJs display a robust form of activity-dependent presynaptic plasticity mediated by increased spontaneous release after high frequency stimulation (HFS). Optical imaging of spontaneous SV releases rates are shown before and after stimulation. Note the different y-axis scale on the post-stimulation map. **b** The enhanced spontaneous release observed at control NMJs after stimulation is abolished in *Syt4* mutants. Arrows denote the timing of HFS to the motor axon. **c** Screen for muscle RNAi knockdown of loci that disrupt postsynaptic membrane expression of a SYT4-pHlourin (SYT4-pH) construct. Control SYT4-pH localization to the postsynaptic membrane is shown on the left. *Syx4*‐RNAi reduces membrane SYT4-pH and causes a redistribution of the protein to cytoplasmic puncta within the muscle (right). The motoneuron is stained with anti-HRP (red). **a** Modified from [95], **b** modified from [104], and **c** modified from [105]

Several synaptic phenotypes were identified in *Syt4* mutants. Drosophila NMJs display a unique form of presynaptic plasticity following high frequency stimulation. Within a minute after strong stimulation of the motor nerve, a robust increase in the frequency of spontaneous release is observed (Fig. 6a) [95, 97, 104, 106, 309]. This enhanced mini rate lasts for several minutes and requires postsynaptic Ca^2+^ influx to trigger release of a retrograde signal from the muscle that increases cAMP levels in presynaptic boutons [104]. The elevation in spontaneous release requires presynaptic PKA phosphorylation of CPX to reduce its clamping function [95]. This activity-dependent increase in spontaneous release is eliminated in *Syt4* mutants (Fig. 6b) [104], suggesting SYT4 is either required to directly mediate release of an unknown retrograde signal or to enable the process through other mechanisms. In addition to the acute effects on presynaptic release, postsynaptic Ca^2+^ influx also drives SYT4-dependent retrograde signaling that promotes synaptic differentiation and synaptic growth [97, 104, 105]. Embryos lacking SYT4 have delayed synapse formation and mutant 3rd instar larvae have fewer synaptic boutons [97, 104]. *Syt4* mutants also disrupt activity-dependent formation of “ghost” boutons that represent nascent presynaptic outgrowths that lack postsynaptic specializations that rapidly form in response to exceptionally strong presynaptic stimulation [106]. SYT4 overexpression in postsynaptic muscles could also induce overgrowth of presynaptic terminals [97]. Together, these data suggested Ca^2+^-dependent retrograde vesicular trafficking supported by SYT4 induced acute changes in synaptic output and triggered synapse-specific growth, providing a link between short-term synaptic plasticity and activity-dependent synaptic rewiring.

The initial studies of SYT4 function assumed *Syt4* mRNA was transcribed and the protein produced in postsynaptic muscles. A major surprise in the field was the discovery that *Syt4* is only transcribed in presynaptic motoneurons and that the SYT4 protein is transmitted to muscles through the release of presynaptic exosomes [106]. Indeed, endogenously CRISPR-tagged SYT4-GFP expression is eliminated from the postsynaptic compartment following RNAi knockdown of the *Syt4* gene presynaptically but not postsynaptically. SYT4 is one of the several proteins that undergo *trans*-synaptic delivery in extracellular vesicles through a pathway regulated by presynaptic Rab11 [107, 310, 311]. This finding parallels trans-synaptic delivery of Drosophila and mammalian ARC proteins that regulate synaptic plasticity [312–315]. Such a mechanism could allow presynaptic activity to regulate the levels of local retrograde signaling by controlling delivery of essential postsynaptic components required for the process. However, the observation generated a host of new questions on how SYT4 is loaded into exosomes and delivered. In addition, how the protein is unpackaged in the postsynaptic cell and what membrane compartment it ultimately occupies are unknown. Although the original model envisioned SYT4-dependent fusion of a vesicular compartment with the postsynaptic membrane that released retrograde signals, SYT4’s presence in exosomes provided other candidate mechanisms for how it might contribute to retrograde signaling.

To identify components of the SYT4 retrograde signaling pathway that function in the postsynaptic compartment, rather than in presynaptic exosome delivery, a UAS-SYT4-pHlourin (SYT4-pH) construct was generated and expressed directly in postsynaptic cells with a muscle GAL4 driver to visualize its trafficking to the plasma membrane [105]. A targeted screen for muscle genes that disrupted postsynaptic surface expression of SYT4-pH using RNAi knockdown was then performed. This approach identified multiple candidates that altered SYT4 membrane delivery, including the postsynaptic t-SNARE SYX4 (Fig. 6c) [105]. Generation of *Syx4* mutants in Drosophila showed the protein was required for delivery of both SYT4 and the well-known postsynaptic transmembrane protein Neuroligin1 (NLG1) to the postsynaptic membrane. *Syx4* mutants displayed reduced synaptic growth and a loss of activity-induced enhancement of spontaneous release [105], similar to *Syt4* mutants [95, 97, 104, 305]. These findings suggested NLG1 might function within the SYT4 signaling pathway, with alterations in postsynaptic membrane content rather than release of diffusible retrograde signals mediating trans-synaptic communication. Beyond SYX4, several interactors that disrupted postsynaptic surface expression of SYT4-pH were known regulators of endocytosis. Given this data, one hypothesis is SYT4′s role in retrograde signaling is not to trigger release of a diffusible retrograde signal, but rather to endocytose or deliver a postsynaptic membrane protein in a Ca^2+^-regulated manner that initiates a presynaptic response. Similar to SYT1, SYT4 interacts with known regulators of endocytosis like AP-2 [98, 177, 292]. Although these studies found SYX4 and SYT4 act in a common pathway, SYX4 also supported other forms of retrograde signaling that negatively regulated neurotransmitter release by decreasing Ca^2+^ channel abundance and Ca^2+^ release cooperativity at presynaptic AZs [316]. These phenotypes were not observed in *Syt4* mutants, indicating the t-SNARE SYX4 supports additional postsynaptic trafficking steps independent of SYT4 in Drosophila.

SYX4 is the sole *Drosophila* homolog of the mammalian SYX3 and SYX4 postsynaptic t-SNARE subfamily [105, 235, 316]. Postsynaptic vesicle trafficking is also regulated by Ca^2+^ in mammals, with postsynaptic SNAREs and SYTs controlling long-term potentiation (LTP) and activity-dependent AMPA receptor trafficking [97, 104, 105, 143–145, 148, 171, 299–302, 305, 317–326]. Mammalian homologs of SYT4 and SYX3/SYX4 have been implicated in multiple postsynaptic membrane-trafficking steps in dendrites, including regulating exocytosis of the neurotrophin BDNF [143–145, 148, 171, 319, 321–323, 325, 327, 328]. These data suggest SYT4, along with SYX3 and SYX4, may represent a conserved pathway that contributes to membrane trafficking and retrograde signaling within postsynaptic compartments. Beyond retrograde trafficking, mammalian SYT4 and SYT11 have been implicated in other trafficking steps that have not been characterized in Drosophila, including DCV maturation and release, DCV fusion pore dynamics, endosomal trafficking and endocytosis [134, 138, 139, 143, 171, 239, 323, 326, 329].

Going forward, many open questions remain for how SYT4 regulates trafficking of membrane organelles at synapses. One major question surrounds the role of Ca^2+^ binding. Unlike the lack of Ca^2+^ binding by mammalian SYT4 and SYT11, Drosophila SYT4 binds Ca^2+^ and its C2 domain Ca^2+^ binding properties are essential for retrograde signaling [97, 104, 298]. These differences are quite perplexing, as Drosophila SYT4 contains the conserved aspartate to serine substitution in the key D3 residue that defines this family, yet is still able to bind Ca^2+^. Mutation of this residue back to an aspartate in the mammalian homologs restore Ca^2+^ binding, indicating the on/off nature of this interaction is tied specifically to this residue [294]. In addition, all the remaining Ca^2+^ binding aspartate residues in C2A and C2B are conserved across evolution in the SYT4 family, unlike the higher divergence seen in other SYT family members that act through Ca^2+^ independent mechanisms. Finally, membrane organelles containing mammalian SYT4 or SYT11 can undergo Ca^2+^-regulated fusion [171, 323]. These findings suggest a potential in vivo ability of mammalian SYT4 and SYT11 to bind Ca^2+^ that is not captured in in vitro studies. One possibility is that the serine residue in the Ca^2+^ binding pocket mediates lower affinity Ca^2+^ interactions that are difficult to detect in vitro. Another option is the serine residue is a site of phosphorylation that would reintroduce stronger negative charge in the Ca^2+^ binding pocket, allowing SYT4 to function in a phosphorylation-controlled manner in vivo. Mutagenesis of this serine in Drosophila has demonstrated the residue is critical for SYT4 function in vivo [97]. The specific conservation of serine at this site in the SYT4 family across ~ 800 million years of evolution suggests it has functional significance, but more work is required to define its role in supporting Ca^2+^ binding in vivo.

Additional questions on SYT4 function revolve around the discovery that the protein is found on exosomes in Drosophila. There are no current studies indicating the mammalian homologs are present on exosomes. SYT4 positive compartments co-localize with DCV markers and BDNF in mammals [143, 171, 330], while BDNF has been found presynaptically and in exosomes from brain extracts [331–333]. It is currently unclear if SYT4 will emerge as a common component of exosomes similar to ARC, or if this delivery mechanism is unique to Drosophila. There is also much to be done on how SYT4 is loaded and unloaded from exosomes. Given the requirement for SYT4 C2 domain interactions, it seems likely these domains would orient towards the cytoplasm within the postsynaptic compartment to bind Ca^2+^ and regulate trafficking. How SYT4-containing exosomes transit the synaptic cleft and are taken up by the postsynaptic cell is unknown. Once inside the muscle, how do SYT4 proteins transit to a specific postsynaptic compartment to regulate synaptic plasticity? What potential retrograde signaling molecules are present in these compartments? Alternatively, does SYT4 act primarily at the postsynaptic membrane to control endocytosis of trans-synaptic signaling components? Future work on this family should provide important insights into similarities between Ca^2+^-regulated trafficking steps in pre- versus post-synaptic compartments.

## Role of the remaining SYT isoforms

Beyond the abundant SYT family members (SYT1, SYT4, SYT7), four additional *Syt* genes are expressed at lower levels or in specific neuronal subpopulations in Drosophila [99, 100]. Syt-α and Syt-β contain highly conserved C2 domain Ca^2+^ binding residues that indicate a role as Ca^2+^ sensors. Antisera raised against the two proteins demonstrate they are expressed in multiple neuropeptide releasing neurons in both the CNS and PNS [100]. Syt-α and Syt-β expression are also controlled by a master regulator of neuroendocrine neurons, the Dimmed transcription factor [108–110, 334–336]. These data are consistent with Syt-α and Syt-β residing on DCVs to mediate Ca^2+^-dependent release of neuropeptides, though genetic analysis will be required to define their function. It is unclear if the more abundant SYT isoforms (SYT1, SYT4, SYT7) also serve as Ca^2+^-sensors for DCV fusion in Drosophila, given their established role for this step in many mammalian neurons. With the limited expression of Syt-α and Syt-β in specific neuronal subpopulations, and the presence of DCVs in most Drosophila neurons, it is likely the more abundant SYT isoforms also participate in DCV trafficking and fusion.

The remaining two SYT isoforms, SYT12 and SYT14 lack conserved Ca^2+^ binding residues and are unlikely to function as Ca^2+^ sensors. RNA profiling and in situ analysis indicate these genes are expressed at very low levels (Fig. 3b). *Syt12* mRNA was not detected using in situ to Drosophila embryos, while RNA profiling showed low expression in the larval and adult brain. One study of mammalian SYT12 found it resides on SVs and acts as a PKA substrate to control spontaneous SV release by multimerizing with SYT1 and negatively regulating its function [337]. Knockout *Syt12* mice are viable and show no changes in baseline synaptic transmission [338]. A disruption of presynaptic cAMP-dependent mossy-fiber LTP in the hippocampal CA3 region was the only defect found in *Syt12* mutants, suggesting a link to PKA regulation of release in this brain area. It is unlikely Drosophila SYT12 is a ubiquitous component of SVs given the low abundance, but it may regulate membrane trafficking in a small population of CNS neurons. The Drosophila *Syt14* gene is present at low levels throughout the embryonic CNS based on in situ analysis [100]. RNA profiling found low to moderate expression of *Syt14* in the CNS of larvae and adults as well, with weaker expression in salivary glands and testis. The only link to SYT14 function comes from a single report suggesting *Syt14* mutations may cause autosomal recessive spinocerebellar ataxia in humans [339]. Further work will be required to define the expression patterns and subcellular locations for these remaining SYT proteins and determine if and how they participate in membrane trafficking in *Drosophila* neurons.

## Relevance of Synaptotagmin dysfunction to human disease

Specific mutations in several SYT family members have been identified in human neurological disorders that affect both the CNS and PNS. In addition, several SYTs are linked more loosely to pathogenesis in neurological disorders arising from other primary causes. The first mutations identified in human SYTs were found in the SYT2 C2B domain in patients presenting with non-progressive peripheral motor neuropathies with unusual similarities to Lambert–Eaton myasthenic syndrome (LEMS) [189, 190, 340, 341]. LEMS is an autoimmune disorder commonly linked to auto-antibodies against the presynaptic Ca^2+^ channel that disrupt NMJ function [342]. SYT2 mutations disrupt motor function and cause peripheral muscle weakness due to defective acetylcholine release at NMJs, but have no obvious effect on cognition. The phenotypes are consistent with data from mice indicating SYT2 is abundantly expressed in the PNS, with SYT1 mediating SV fusion for the majority of CNS neurons [101]. This group of human SYT2 mutations are dominantly inherited and run through family lineages in patient populations, with an affected individual harboring one normal copy and one mutated version of SYT2. All mutations identified to date cluster in specific residues lining the C2B Ca^2+^ binding pocket, suggesting they are likely to dominantly disrupt C2B domain interactions with membrane lipids. Loss of a single copy of the orthologous SYT1 protein does not dramatically alter neurotransmitter release in *Drosophila* or mouse models, suggesting the phenotype is not due to haplo-insufficiency but rather acts by poisoning the fusion machinery [49, 62, 75, 156, 189, 340]. Indeed, homozygous mutations in human SYT2 resulting in complete biallelic loss of the protein have been identified in five families that result in a more severe congenital myasthenic syndrome than in autosomal dominant patients [343]. In this study, heterozygous carries of SYT2 null alleles had no obvious phenotypes, confirming dominant mutations in SYT2 patients are not caused by a simple loss of function haplo-insufficiency model. The less severe phenotypes in patients harboring SYT2 dominant mutations indicate SYT2 function is not abolished in these patients. In addition, SYT1 may play a partially redundant role in acetylcholine release at NMJs, consistent with phenotypes in *Syt2* knockout mice [101].

Recently, similar heterozygote dominant mutations in the C2B Ca^2+^ binding site of human SYT1 have been identified, resulting in a severe neurodevelopmental disorder with infant hypotonia, hyperkinetic movement disorder, profound developmental delay, cognitive dysfunction, and failure to develop language [344, 345]. These mutations are not heritable due to their phenotypic severity and instead arise as spontaneous mutations in the germline. Similar to SYT2 autosomal dominant mutations, these patients maintain residual synaptic transmission, as complete loss of SYT1 function would be lethal. From a clinical perspective, it is essential to define how these dominant-negative C2B mutations disrupt the fusion machinery and to identify potential mechanisms to increase SV release in these backgrounds to bypass the disruption. Future work will be required to determine if the C2B Ca^2+^-binding pocket, the location of all mutations identified to date, is a privileged site for alleles that can dominantly disrupt SV release.

Drosophila contains a single ortholog of the human SYT1 and SYT2 proteins, and mutations corresponding to several of the dominant-negative disease alleles have been modeled [62, 189, 340, 346]. Given SYT1 mediates all synchronous SV release in Drosophila, the phenotypes of these alleles are expected to be more severe than individual human SYT1 or SYT2 mutants. Indeed, overexpression of several of these alleles, including an aspartate to alanine substitution in the C2B domain D2 Ca^2+^ binding residue, cause lethality when overexpressed in Drosophila [189]. This mutation did not rescue any *Syt1* null phenotypes, indicating the amino acid change completely abolished the ability of the protein to function. How do these mutant SYT1 and SYT2 proteins dominantly interfere with neurotransmitter release? The studies in Drosophila suggest mutant SYT alleles are likely to multimerize with endogenous SYT1 or SYT2 and disrupt the normal function of a SYT oligomer in a dominant fashion. Another model based on studies in mice suggests loss of Ca^2+^-dependent lipid binding is the primary mechanism [347]. How this property alone would trigger a dominant phenotype, given loss of function of a single copy of SYT1 or SYT2 does not disrupt release, is less clear unless the mutant was also able to disrupt the wildtype SYT1 proteins encoded from the non-affected chromosome. As described above, a genetic screen to find second-site mutations that reduce the dominant disruption of SV fusion caused by C2B Ca^2+^ binding mutants was performed in Drosophila that identified interactions required for the pathogenic mechanism [62]. This study found these C2B mutants require oligomerization and SNARE binding, as well as Ca^2+^-independent lipid interactions through the C2B polybasic region, to dominantly poison the endogenous SV fusion machinery. These interactions likely position mutant SYT proteins near release sites where they dominantly disrupt fusion even in the presence of a normal copy of the protein. Two candidate mechanisms for the dominant effect seem most likely. In one model, a defect in the ability of the C2B domain loops to penetrate lipid bilayers upon Ca^2+^ influx would poison the fusion machinery by oligomerizing with the wildtype copy of SYT1. Another possibility is that the disease alleles result in neutralization of negative charge or increased hydrophobicity of the C2B loops, resulting in spurious activation of Ca^2+^-independent lipid interactions by the mutant protein. This could bring a SYT oligomer to membranes at inappropriate times, or act to clog release sites as a monomer only that blocks the activity of the normal allele. With genome sequencing becoming more common, it is likely more cases of SYT-mediated neurological disorders will be found given a disruptive amino acid change in the C2B Ca^2+^-binding pocket in a single copy can cause disease.

Beyond neurological disorders caused by SYT1 and SYT2 mutations, the remaining links to disease are still somewhat speculative and relatively understudied. Disruptions of SYT7 have been suggested as a risk factor for bipolar disorder (BD) in humans, with mutant *Syt7* mice displaying manic and depression like behaviors [251, 348]. A study of BD patients showed reduced plasma levels of *Syt7* mRNA, and several SNPs in the *Syt7* gene have been identified in BD individuals [251, 348]. SYT7 proteins levels are also decreased in some variants of Alzheimer’s disease [349] and a role in lysosomal trafficking and membrane repair is implicated in muscular dystrophy [261, 262]. A few studies have associated SYT11 with schizophrenia and Parkinson’s disease (PD), and SYT11 can accumulate in dopaminergic neurons lacking Parkin [350–353]. SYT14 mutations have been reported to cause autosomal recessive spinocerebellar ataxia with psychomotor retardation in humans [339, 354], but little else is known about the protein. Dysregulation of expression of several SYTs have also been reported in models of neurodegenerative disorders, including Parkinson’s, Alzheimer’s, and Amyotrophic Lateral Sclerosis (ALS) [355–367]. Overall, the mechanisms by which other SYT isoforms participate in disease pathogenesis is largely unknown. However, given the broad role of SYT proteins in regulating intracellular membrane trafficking and fusion within neurons, it is likely more links to brain diseases will be identified going forward.
